# Supplementary material for: Human-specific evolutionary markers linked to foetal neurodevelopment modulate brain surface area in schizophrenia
Source: Commun Biol. 2023 Oct 13;6:1040. doi: 10.1038/s42003-023-05356-2 (PMC10576001; doi:10.1038/s42003-023-05356-2)
Supplement: Supplementary file 2 — Description of additional supplementary files [file 42003_2023_5356_MOESM2_ESM.docx]

**Supplementary Data Tables description**

1) Supplementary_Data_1: PRSs effect on patients with SZ clinical profile.

2) Supplementary_Data_2: PRSs models on cortical thickness (CT) and surface area (SA).

3) Supplementary_Data_3: Surface Area (SA) effect on patients with SZ clinical profile.

4) Supplementary_Data_4: FUMA SNP2GENE annotation results for FB-HARs PRSsz SNPs.

5) Supplementary_Data_5: FUMA GENE2FUNC annotation results for FB-HARs PRSsz SNPs.

6) Supplementary_Data_6: 3,070 autosomic HAR regions compiled by Girskis et al., 2021.

7) Supplementary_Data_7: HARs SNPs sets for the PRSs estimations.

8) Supplementary_Data_8: Data on the four estimated PRSs.

9) Supplementary_Data_9: Numerical source data for Figure 1.
