# Supplementary material for: Human-specific evolutionary markers linked to foetal neurodevelopment modulate brain surface area in schizophrenia
Source: Commun Biol. 2023 Oct 13;6:1040. doi: 10.1038/s42003-023-05356-2 (PMC10576001; doi:10.1038/s42003-023-05356-2)
Supplement: Supplementary file 4 — Reporting Summary [file 42003_2023_5356_MOESM4_ESM.pdf]

nature portfolio

Corresponding author(s): Maria Guardiola-Ripoll & Mar Fatjó-Vilas, COMMSBIO-23-0926A

Last updated by author(s): Aug 31, 2023

Reporting Summary

Nature Portfolio wishes to improve the reproducibility of the work that we publish. This form provides structure for consistency and transparency in reporting. For further information on Nature Portfolio policies, see our Editorial Policies and the Editorial Policy Checklist.

Statistics

For all statistical analyses, confirm that the following items are present in the figure legend, table legend, main text, or Methods section.

n/a	Confirmed	
☐	☒	The exact sample size (*n*) for each experimental group/condition, given as a discrete number and unit of measurement
☐	☒	A statement on whether measurements were taken from distinct samples or whether the same sample was measured repeatedly
☐	☒	The statistical test(s) used AND whether they are one- or two-sided<br>*Only common tests should be described solely by name; describe more complex techniques in the Methods section.*
☐	☒	A description of all covariates tested
☐	☒	A description of any assumptions or corrections, such as tests of normality and adjustment for multiple comparisons
☐	☒	A full description of the statistical parameters including central tendency (e.g. means) or other basic estimates (e.g. regression coefficient) AND variation (e.g. standard deviation) or associated estimates of uncertainty (e.g. confidence intervals)
☐	☒	For null hypothesis testing, the test statistic (e.g. *F*, *t*, *r*) with confidence intervals, effect sizes, degrees of freedom and *P* value noted<br>*Give P values as exact values whenever suitable.*
☒	☐	For Bayesian analysis, information on the choice of priors and Markov chain Monte Carlo settings
☒	☐	For hierarchical and complex designs, identification of the appropriate level for tests and full reporting of outcomes
☐	☒	Estimates of effect sizes (e.g. Cohen's *d*, Pearson's *r*), indicating how they were calculated

Our web collection on statistics for biologists contains articles on many of the points above.

Software and code

Policy information about availability of computer code

Data collection	Demographic and clinical data: SPSS v29.00. Genotyping: Infinium Global Screening Array-24 v1.0 (GSA) BeadChip, re-phasing and imputation: Eagle, Minimac4 and the Haplotype Reference Consortium dataset (HRC version r1.1) (Michigan Imputation Server). PRS estimation: PLINK 1.90. MRI adquisition: 1.5T GE Sigma and 3T Philips Ingenia scanners. Surface-based morphometry: FreeSurfer.
Data analysis	SPSS v29.0 and R software. SNP and gene annotation: FUMA.

For manuscripts utilizing custom algorithms or software that are central to the research but not yet described in published literature, software must be made available to editors and reviewers. We strongly encourage code deposition in a community repository (e.g. GitHub). See the Nature Portfolio guidelines for submitting code & software for further information.

Data

Policy information about availability of data

All manuscripts must include a data availability statement. This statement should provide the following information, where applicable:

- Accession codes, unique identifiers, or web links for publicly available datasets
- A description of any restrictions on data availability
- For clinical datasets or third party data, please ensure that the statement adheres to our policy

The data that support the findings of this study are available from the corresponding authors upon reasonable request.

Research involving human participants, their data, or biological material

Policy information about studies with human participants or human data. See also policy information about sex, gender (identity/presentation), and sexual orientation and race, ethnicity and racism.

Reporting on sex and gender	The sample of this study was assessed based on sex (biological attribute). A group matching procedure was conducted to minimise age and sex differences across diagnostic groups while maximising the sample size.
Reporting on race, ethnicity, or other socially relevant groupings	In order to avoid population stratification due to differences in the allelic distributions, the studied was conducted in an homogenous group of participants of European ancestry.
Population characteristics	All participants were between 18 and 65 years old, right-handed and had an estimated intelligence quotient (IQ) (premorbid IQ in patients), higher than 70, as assessed using the Spanish version of the Word Accentuation Test. All participants met the same exclusion criteria: suffering from major medical illness, conditions affecting cognitive or brain function, neurological conditions, history of head trauma with loss of consciousness and present or history of drug abuse or dependence. Additionally, for healthy controls, exclusion criteria also included personal or family history of psychiatric service contact or treatment.
Recruitment	Patients were recruited from different centres of Germanes Hospitalaries in the area of Barcelona province and healthy controls from the same area.
Ethics oversight	Germanes Hospitalàries Research Ethics Committee. Procedures and implications in accord with the Declaration of Helsinki.

Note that full information on the approval of the study protocol must also be provided in the manuscript.

Field-specific reporting

Please select the one below that is the best fit for your research. If you are not sure, read the appropriate sections before making your selection.

☒ Life sciences ☐ Behavioural & social sciences ☐ Ecological, evolutionary & environmental sciences

For a reference copy of the document with all sections, see nature.com/documents/nr-reporting-summary-flat.pdf

Life sciences study design

All studies must disclose on these points even when the disclosure is negative.

Sample size	No sample-size calculation was performed. These neuroimaging genetic analyses have been conducted in a sample of hundreds of individuals, exceeding the median sample size of neuroimaging association studies according to a recent revision (Marek et al. 2022).
Data exclusions	A group matching procedure was conducted to minimise the differences across diagnostic groups while maximising the sample size. The analyses were conducted in a sample of 115 healthy controls and 128 patients with a schizophrenia diagnosis with no age and sex differences.
Replication	There was an insufficient sample to conduct replication analyses, and therefore the findings have not been replicated.
Randomization	Not applicable.
Blinding	Not applicable.

Reporting for specific materials, systems and methods

We require information from authors about some types of materials, experimental systems and methods used in many studies. Here, indicate whether each material, system or method listed is relevant to your study. If you are not sure if a list item applies to your research, read the appropriate section before selecting a response.

Materials & experimental systems

n/a	Involved in the study	
☒	☐	Antibodies
☒	☐	Eukaryotic cell lines
☒	☐	Palaeontology and archaeology
☒	☐	Animals and other organisms
☐	☒	Clinical data
☒	☐	Dual use research of concern
☒	☐	Plants

Methods

n/a	Involved in the study	
☒	☐	ChIP-seq
☒	☐	Flow cytometry
☐	☒	MRI-based neuroimaging

Clinical data

Policy information about clinical studies

All manuscripts should comply with the ICMJE guidelines for publication of clinical research and a completed CONSORT checklist must be included with all submissions.

Clinical trial registration	Not applicable.
Study protocol	Not applicable.
Data collection	The clinical data assessed in the study includes the diagnostic status (patients with schizophrenia vs. healthy controls) and a neurocognitive assessment (Spanish version of the Word Accentuation Test). Specifically for patients, the illness duration (estimated based on the dete of first contact with psychiatric services and the MRI date), symtomatology, as asssessed using the Positive and Negative Symptoms Scale (PANSS) and medication dose (using chlorpromazine equivalent dose) were also assessed.
Outcomes	Premorbid IQ, Ilness duration (in years), PANSS scores, and chlorpromazine equivalent dose (in mg/day).

Magnetic resonance imaging

Experimental design

Design type	Case-control study on surface-based morphometry.
Design specifications	Structural MRI data were processed using the FreeSurfer image analysis suite (http://surfer.nmr.mgh.harvard.edu/). Image pre-processing included removal of non-brain tissue, automated Talairach transformation, tessellation of the grey and white matter boundaries and surface deformation 64. Several deformation procedures were performed in the data analysis pipeline, including surface inflation and registration to a spherical atlas. This method uses both intensity and continuity information from the entire three-dimensional images in the segmentation and deformation procedures to produce vertex-wise representations of cortical thickness (CT) and surface area (SA). The CT was defined as the measure of the distance between the white matter surface and the pial surface, and cortical SA was calculated as the area of the white matter surface. With FreeSurfer, we automatically performed the segmentation of 34 cortical regions of interest for each hemisphere using the Desikan-Killiany cortical atlas. Mean values of CT and SA were quantified for each individual within these defined regions
Behavioral performance measures	Not applicable.

Acquisition

Imaging type(s)	High-resolution structural-T1 MRI data.
Field strength	1.5T GE Sigma scanner and 3T Philips Ingenia scanner.
Sequence & imaging parameters	High-resolution structural-T1 MRI data in the 1.5T scanner were obtained using the following acquisition parameters: matrix size 512 x 512; 180 contiguous axial slices; voxel resolution 0.47 x 0.47 x 1 mm^3; echo time (TE) = 3.93 ms, repetition time (TR) = 2,000 ms; and flip angle = 15º. At the 3T scanner, high resolution structural-T1 MRI data were obtained with the following acquisition parameters: matrix size 320 × 320 x 250; voxel resolution 0.75 x 0.75 x 0.80 mm^3; TE= 3.80 ms, TR = 8.40 ms; and flip angle = 8º. All images were visually inspected to exclude those with artefacts and movement.
Area of acquisition	Whole brain scan.

Diffusion MRI ☐ Used ☒ Not used

Preprocessing

Preprocessing software	Structural MRI data were processed using the FreeSurfer image analysis suite.
Normalization	Image pre-processing included removal of non-brain tissue, automated Talairach transformation, tessellation of the grey and white matter boundaries and surface deformation. Several deformation procedures were performed in the data analysis pipeline, including surface inflation and registration to a spherical atlas. This method uses both intensity and continuity information from the entire three-dimensional images in the segmentation and deformation procedures to produce vertex-wise representations of Cortical Thickness and Surface Area. The normalization was carried out using the standard approach as implemented in FreeSurfer.
Normalization template	Registration of a subject's surface to a reference consists of a 2-D warp of the subject's sphere surface so that the individual's curvature data pattern aligns with a reference template pattern. The template was previously prepared as the "average" pattern from a group of representative subjects.
Noise and artifact removal	Not applicable.

Volume censoring | Not applicable.

Statistical modeling & inference

Model type and settings | Regression model.

Effect(s) tested | We applied linear regression models within diagnostic groups (separately in healthy controls and patients with schizophrenia) to test the effect of each polygenic risk score (PRS) on Cortical Thickness and Surface Area. To assess whether the PRS effect was modulated by the diagnostic status, we conducted linear models using the whole sample and tested the PRS x diagnosis interaction.

Specify type of analysis: ☐ Whole brain ☒ ROI-based ☐ Both

Anatomical location(s) | ROI-based analyses on 34 cortical regions of interest for each hemisphere using the Desikan-Killiany cortical atlas (Desikan et al., 2006)

Statistic type for inference | Not applicable.

(See Eklund et al. 2016)

Correction | The p-values resulting from each one of the before mentioned statistical tests were adjusted by using the false discovery rate (FDR) method, specifically the Benjamini-Hochberg procedure, to control for multiple comparisons at level q=0.05. Accordingly, only those results with a corrected FDR-pval<0.05 were considered statistically significant.

Models & analysis

n/a	Involved in the study
☒	☐ Functional and/or effective connectivity
☒	☐ Graph analysis
☒	☐ Multivariate modeling or predictive analysis
